# A novel synaptic junction preparation for the identification and characterization of cleft proteins

**DOI:** 10.1371/journal.pone.0174895

**Published:** 2017-03-31

**Authors:** Amelia Burch, Jung-Hwa Tao-Cheng, Ayse Dosemeci

**Affiliations:** 1 Laboratory of Neurobiology, National Institute of Neurological Disorders and Stroke, National Institutes of Health, Bethesda, Maryland, United States of America; 2 EM Facility, National Institute of Neurological Disorders and Stroke, National Institutes of Health, Bethesda, Maryland, United States of America; University of Michigan, UNITED STATES

## Abstract

Identification of synaptic cleft components has been hampered by the lack of a suitable preparation enriched in synaptic junctions devoid of adjoining peripheral membranes. Prior strategies for the isolation of synaptic junctions, relying on detergents for the removal of peripheral membranes, resulted in substantial loss of membranes lining the cleft. Here, a novel, detergent-free method is described for the preparation of a synaptic junction (SJ) fraction, using phospholipase A_2_. Limited digestion of synaptic plasma membrane (SPM) fraction with phospholipase A_2_ followed by centrifugation over a sucrose cushion results in selective removal of membranes peripheral to the cleft while junctional membranes remain relatively intact as observed by electron microscopy. Enrichment in synaptic junctional structures and loss of membranes peripheral to the junctional area are further verified by demonstrating enrichment in PSD-95 and loss in mGluR5, respectively. The SJ fraction is enriched in neuroligins and neurexins, in agreement with immuno-electron microscopy data showing their selective localization to the junctional area. Among additional cell adhesion molecules tested, N-cadherin and specific isoforms of the SynCAM and SALM families also show marked enrichment in the SJ fraction, suggesting preferential localization at the synaptic cleft while others show little enrichment or decrease, suggesting that they are not restricted to or concentrated at the synaptic cleft. Treatment of the SJ fraction with glycosidases results in electrophoretic mobility shifts of all cell adhesion molecules tested, indicating glycosylation at the synaptic cleft. Biochemical and ultrastructural data presented indicate that the novel synaptic junction preparation can be used as a predictive tool for the identification and characterization of the components of the synaptic cleft.

## Introduction

The synaptic cleft is a ~20 nm gap between pre- and postsynaptic compartments [1]. Structures that traverse the cleft from the pre- to the postsynaptic membrane are revealed by electron microscopy (EM) [2], [3]. A recent study, using freeze substitution and EM tomography, identified distinct types of these trans-synaptic structures [4]. The structures bridging the cleft are likely formed by synaptic cell adhesion molecules originating from the pre- and postsynaptic sites, respectively. These molecules have key roles in synaptic adhesion and also act as organizing and signaling elements [5].

**Fig 1:**
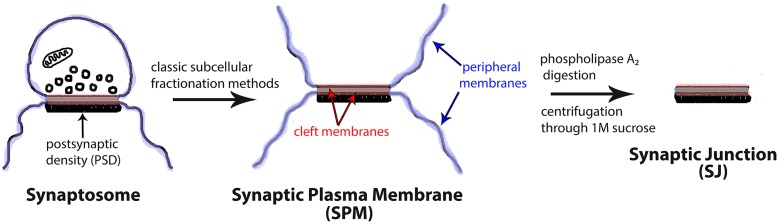
Strategy for the isolation of synaptic junctions. The synaptic cleft is highlighted in gray. ‘Cleft membranes’ are defined as the membranes within the synaptic junctional area, highlighted in red. Membranes peripheral to the synaptic junction are referred to as ‘peripheral membranes’ and are highlighted in blue. Treatment of the SPM fraction with phospholipase A_2_ is expected to promote preferential removal of peripheral membranes as compared to the relatively occluded cleft membranes.

A fundamental criterion for the classification of proteins as synaptic cell adhesion molecules is localization to the synaptic cleft membranes [5]. Typically, cell adhesion molecules are classified as synaptic cell adhesion molecules if they co-localize with synaptic markers by immunofluorescence microscopy or co-purify with synaptosomes or synaptosome-derived fractions. While these approaches have been instrumental in revealing several potential cleft components, they can also lead to erroneous classifications due to the inability to differentiate between synaptic cleft membranes and membranes peripheral to the cleft (Fig 1).

Recently, Loh *et al* applied an alternative strategy, based on spatially restricted enzymatic tagging, for the identification of molecules at the synaptic cleft [6]. The resulting list of proteins indeed contains several *bona fide* cleft components whose localization had been verified ultrastructurally. However, also included in the list are molecules such as metabotropic glutamate receptors of group I (gene name *GRM1*) known to be localized to the peripheral membranes [7], [8], [9], again, indicating the possibility of false positives.

Isolation of a fraction enriched in synaptic junctions (SJ) without attached peripheral membranes could be a powerful additional tool for the identification of cleft components. However, prior biochemical methods to isolate synaptic junctions by treatment of synaptic plasma membrane (SPM) fractions with low concentration of TritonX-100 resulted in extensive loss of cleft membranes [10], [11], [12]. The present study describes a novel, detergent-free method for the isolation of synaptic junctions, using phospholipase A_2_, an enzyme which disrupts the phospholipid bilayer. Given that membranes lining the ~20 nm wide synaptic cleft may be shielded by trans-synaptic complexes and other cleft components, phospholipase A_2_, a protein, seemed less likely to penetrate this region compared to Triton X-100, a small molecule with detergent properties. Thus, it was predicted that phospholipase A_2_ may selectively digest the relatively unprotected, peripheral membranes adjacent to the cleft.

## Materials and methods

### Antibodies

Antibodies used are listed in Table 1.

**Table 1:** List of antibodies.

Protein	Antibody	Dilution for Western	Dilution for EM
Neuroligin 1	Synaptic Systems Cat # 129 003 Rabbit polyclonal	1 μg/mL	
	NeuroMab clone N97A/31 Mouse monoclonal	3.3 μg/mL	
Neuroligin 2	Synaptic Systems Cat # 129 511 Mouse monoclonal	1 μg/mL	
Neuroligin 3	Synaptic Systems Cat # 1293211 Mouse monoclonal	1 μg/mL	
	NeuroMab Clone N110/29 Mouse monoclonal	3.3 μg/mL	
Neuroligin 1/2/3/4	Synaptic Systems Cat # 129 211 Mouse monoclonal		1 μg/mL
Neurexin 1/2/3	Synaptic Systems Cat # 175 003 Rabbit polyclonal	2 μg/mL	20 μg/mL
SALM3	Synaptic Systems Cat # 294 303 Rabbit polyclonal	5 μg/mL	
SALM4	Synaptic Systems Cat # 294 403 Rabbit polyclonal	5 μg/mL	
SALM5	Abcam Cat # ab106370 Rabbit polyclonal	1:200	
N-cadherin	Abcam Cat # ab18203 Rabbit polyclonal	1 μg/mL	
Eph A4 Receptor	Zymed Cat # 34–7900 Rabbit polyclonal	1.25 μg/mL	
Ephrin-B	Zymed Cat # 37–8100 Mouse monoclonal	2 μg/mL	
NCAM	Sigma Cat # C 9672 Mouse monoclonal	1:1000	1:100
SynCAM 1/2/3	Synaptic Systems Cat # 243 003 Rabbit polyclonal	1 μg/mL	10 μg/mL
PSD-95	New England Peptide Rabbit polyclonal	0.19 μg/mL	3.8 μg/mL
mGluR5	EMD Millipore Cat # AB5675 Rabbit polyclonal	1 μg/mL	

Neurologin 1 and neuroligin 3 antibodies from Synaptic Systems were used in deglycosylation experiments, while neuroligin antibodies from NeuroMab were used to test for enrichment in the SJ fraction. PSD-95 antibody is custom-made and described previously by Yang *et al* [13].

### Subcellular fractionation methods

Brains from 20–25 weeks-old Sprague-Dawley rats were supplied by Rockland Immunochemicals, Inc (Limerick, PA, USA). Animals were subjected to CO_2_ for 1min before decapitation. Brains were collected and flash frozen in liquid nitrogen within 2min of harvest and shipped on dry ice. Upon receipt, brains were kept at -80°C until use. Frozen brains were rapidly thawed by 1min immersion in 0.32M sucrose at 37°C. Cerebral cortices were dissected and immediately homogenized in 0.32 M sucrose, 1 mM MgCl2, 1 μg/ml leupeptin, 1 mM HEPES (pH 7), using a motor-driven glass/teflon homogenizer.

#### Synaptic Plasma Membrane (SPM) preparation

A conventional strategy originally devised by Gray & Whittaker [14] was applied for the preparation of synaptosome and synaptic plasma membrane (SPM) fractions. Samples were kept on ice throughout the protocol and all centrifugation steps were carried out at 4°C. The homogenate was centrifuged at 1,400 g for 10min. The supernatant from this step was saved, and pellets were resuspended in 0.32 M sucrose and centrifuged at 710 g for 10min. The supernatants from the two steps were combined and recentrifuged at 710 g for 10min. The resulting pellets were discarded and the supernatant (S1) was centrifuged at 13,800 g for 10min to obtain the P2 and S2 fractions. The P2 fraction was then layered on a sucrose gradient (0.85M/1M/1.25M). Synaptosomes were collected from the 1M/1.25M interface and subjected to hypotonic lysis (at least 10 X dilution in 1mM HEPES) and centrifuged at 10,528 g for 30min using a fixed angle rotor. Pellets were resuspended in 100mM KCl, 1mM HEPES, layered on a sucrose gradient (0.85M/1M/1.25M) and centrifuged at 200,000 g for 2h using a swinging bucket rotor. The SPM-enriched layer (1/1.2M sucrose interface) was collected and stored at -20°C in 33% glycerol.

#### Synaptic Junction (SJ) preparation

The strategy for the isolation of synaptic junctions is outlined in Fig 1. For optimum results, SPM fractions containing 200 μg protein were incubated with 2 μg (3.6 units) of phospholipase A_2_ from honeybee venom (Sigma, St. Louis, MO, USA, Cat # P9279) in medium containing 1 mM CaCl_2_ and protease inhibitors (ThermoFisher Scientific, Waltham, MA, USA Cat # 78415) in 20mM Tris-HCl, pH 8, in a final volume of 250 μL for 20min at 20°C. The reaction was stopped with addition of ice-cold KCl to yield a final concentration of 0.2M KCl in 1mL total volume. Digested samples were then layered on 500μL of 1.0M sucrose/0.2M KCl cushion and centrifuged at 11,700 g for 1h. The SJ-enriched pellets were collected and stored at -20°C in 33% glycerol. In some experiments, the supernatants (the upper original sample layer + the lower sucrose cushion) were also collected for further analysis. Supernatants were precipitated with 72% trichloroacetic acid and re-solubilized in SDS-containing PAGE sample buffer.

The Bradford method was employed for estimation of protein concentration of fractions, using Bio-Rad Protein Assay Dye Reagent Concentrate (Cat # 5000006).

### Deglycosylation

Deglycosylation was performed according to ‘Denaturation Protocol’ using Sigma’s Enzymatic Protein Deglycosylation Kit (Cat # EDEGLY). SJ fraction (100μg protein) was boiled for 5min in denaturing solution containing SDS and β-mercaptoethanol, cooled to room temperature, and 2.5μL of 15% tritonX-100 was added. The samples were then incubated in 50mM sodium phosphate pH 7, with or without (control) the following enzymes provided by the kit; PNGase F, O-Glycosidase, α-(2→3,6,8,9)-Neuraminidase, β-(1→4)-Galactosidase, β-N-Acetylglucosaminidase for 3h at 37°C.

### Gel electrophoresis and Western immunoblotting

Samples were solubilized in SDS-containing PAGE sample buffer and boiled for 5min. Proteins were separated on 4–15% Mini PROTEAN TGX precast polyacrylamide gels from Bio-Rad (Hercules, CA, USA, Cat # 456–1083). Coomassie staining was for 1h with PageBlue^™^ Protein Staining Solution from Thermo Scientific (Waltham, MA, USA, Cat # 24620). For Westerns, samples were transferred to PVDF membranes using Trans-Blot Turbo Transfer System (1.3A, 25 V, 14min) from Bio-Rad which were then incubated in blocking buffer, primary, and secondary antibodies. Immunoblots were visualized by chemiluminescence (Bio-Rad). The relative enrichment of PSD-95 was estimated as the ratio of peak areas from densitometric scans using ImageJ (Bethesda, MD, USA).

### Electron microscopy

#### Electron microscopy of fractions

SPM and SJ fractions (50μg protein) were pelleted, fixed with 4% glutaraldehyde in 0.1M sodium cacodylate buffer, pH 7.6, overnight. Samples were treated with 1% OsO_4_ in cacodylate buffer for 1h on ice, left overnight in 0.25% uranyl-acetate in acetate buffer, pH 5.0, dehydrated in graded ethanols and embedded in Epoxy resin for thin sectioning.

#### Preparation of hippocampal cell cultures, perfusion-fixed mouse brains, pre-embedding immuno-electron microscopy

The animal protocol was approved by the National Institute of Neurological Disorders and Stroke/National Institute of Deafness and Communications Disorders/National Center for Complementary and Integrative Health Animal Use and Care Committee and conforms to NIH guidelines. Perfusion fixation of mouse brain was performed as previously described in Tao-Cheng *et al* (15). Hippocampi from 20- to 21-day embryonic Sprague-Dawley rats were dissociated and grew on glial cells for 3 weeks as previously described [15]. Cells were fixed in 2–4% paraformaldehyde (EMS, Fort Washington, PA, USA) in PBS for 10-35min, washed in PBS, and stored at 4°C. Cells were permeabilized in either 50% ethanol for 10min and blocked with 5% normal goat serum in PBS for 30min or permeabilized and blocked with 0.1% saponin and 5% normal goat serum in PBS for 30min. Samples were incubated in primary and secondary antibodies (Nano-gold, Nanoprobes, Yaphand, NY, USA) for 1h at room temperature, fixed in 2% glutaraldehyde in PBS, and stored at 4°C. Cell were washed in deionized water, silver-enhanced (HQ kit, Nanoprobes), and processed for EM as described in Tao-Cheng *et al* [15].

## Results

**Fig 2:**
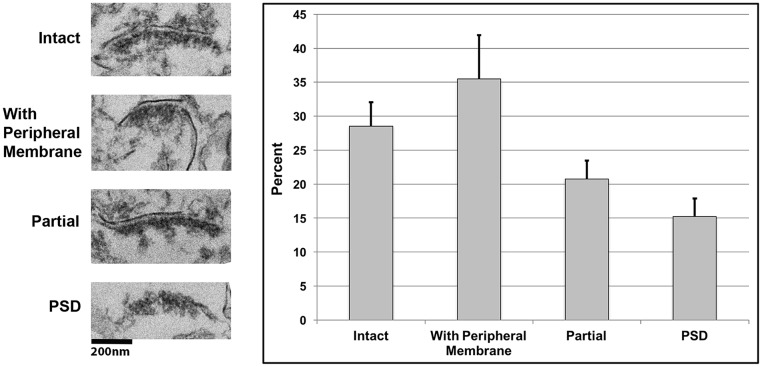
Assessment of the optimized synaptic junction preparation by electron microscopy. Four different synaptic junction fractions were evaluated by electron microscopy. Every recognizable synaptic structure with a postsynaptic density (PSD) was counted and classified into one of the four categories. Ideal synaptic junctions devoid of peripheral membranes and without damage to the cleft membranes were identified as ‘intact’. Synaptic junctions with some undigested peripheral membranes were categorized as ‘with peripheral membranes’. Synapses with some damage to the cleft membranes were identified as ‘partial’, while synapses with total loss of membranes were counted as ‘PSDs’. The bars represent percentages for each type of synaptic structure as a mean of four experiments. The error bars represent the standard error of the mean.

SPM fractions were incubated with phospholipase A_2_, and the samples were then layered on a sucrose cushion and centrifuged to separate lighter membranes from the denser, junctional material. The quality of the synaptic junctional pellets was evaluated by electron microscopy. Every recognizable synaptic structure encountered was scored according to the presence or absence of peripheral membranes and the intactness of cleft membranes (Fig 2, S1 Fig). The SJ preparation protocol was optimized by altering the concentration of phospholipase A_2_ and adjusting the duration of the reaction. Under the optimized conditions (See Methods), the majority of synaptic material displayed intact, junctional structures. Less than 20% of synaptic material was classified as PSD-like structures devoid of synaptic junctional membranes (Fig 2). The fraction also contained some mitochondrial and membrane contaminants.

**Fig 3:**
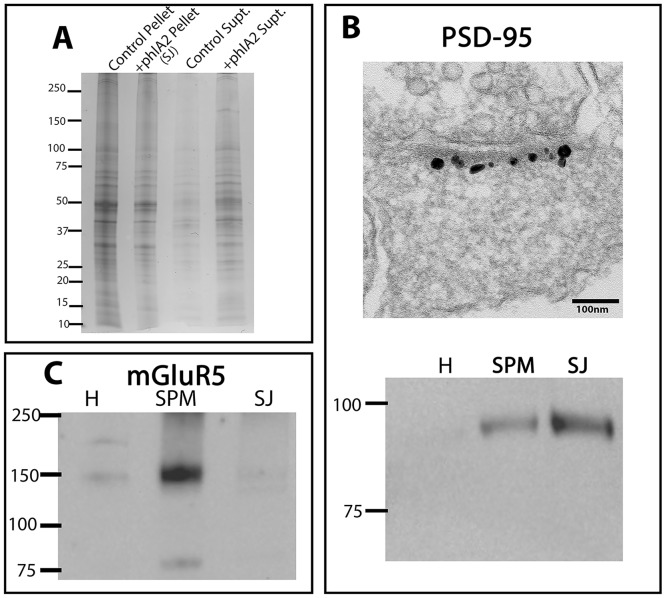
Verification of synaptic junction fraction. **A)** Synaptic plasma membrane (SPM) fractions were incubated in presence (+phlA2) or absence (control) of phosopholipase A_2_ and centrifuged (See Methods). Comparison of Coomassie Blue stained lanes shows dissociation of proteins as a result of phospholipase A_2_ digestion. **B)** Top: Immuno-electron microscopy shows immunogold labeling for PSD-95 localized selectively to the synaptic junction region in cultured rat hippocampal neurons. Bottom: Immunoblots with an antibody for PSD-95 show enrichment of PSD-95 in +phlA2 pellet called ‘synaptic junction’ (SJ) fraction compared to parent homogenate (H) and SPM fractions. Equal amounts of protein were loaded into each lane. The relative enrichment of PSD-95 in the SJ fraction, as compared to that in the parent SPM fraction, was estimated as the ratio of peak areas from densitometric scans. The mean enrichment factor (fold enrichment) from seven immunoblots corresponding to four different SJ preparations was 3.2±0.76. **C)** Immunoblot with an antibody for mGluR5 shows enrichment of mGluR5 in the SPM fraction compared to the parent homogenate fraction, while there are decreased levels of mGluR5 in the SJ fraction compared to parent SPM fraction. Equal amounts of protein were loaded into each lane.

Comparison of pellets and supernatants from phospholipase A_2_-treated and control samples in Coomassie Blue stained gels indicated a substantial amount of protein fractionating into supernatants following phospholipase treatment (Fig 3A). In agreement with these results, protein estimation showed an average recovery of 35±3.3% (five experiments) of the original SPM protein in pellets following phospholipase A_2_ treatment and centrifugation. Removal of peripheral membranes with associated proteins upon phospholipase A_2_ digestion should result in an enrichment of proteins located selectively at the junctional area. PSD-95, a PSD-associated scaffold protein constitutes a good marker for the junctional area as deduced from immuno-EM (Fig 3B, top). Comparison of the pellet after phospholipase A_2_ digestion (SJ fraction) with parent homogenate (H) and SPM fractions indeed indicated substantial enrichment of PSD-95 (Fig 3B, bottom). Verification for the removal of peripheral membranes was provided by tracking the levels of the metabotropic glutamate receptor, mGluR5, known to be localized on peripheral membranes outside the synaptic junction [7], [8], [9]. Indeed, mGluR5 levels were drastically reduced in the SJ fraction compared to the parent SPM fraction (Fig 3C).

**Fig 4:**
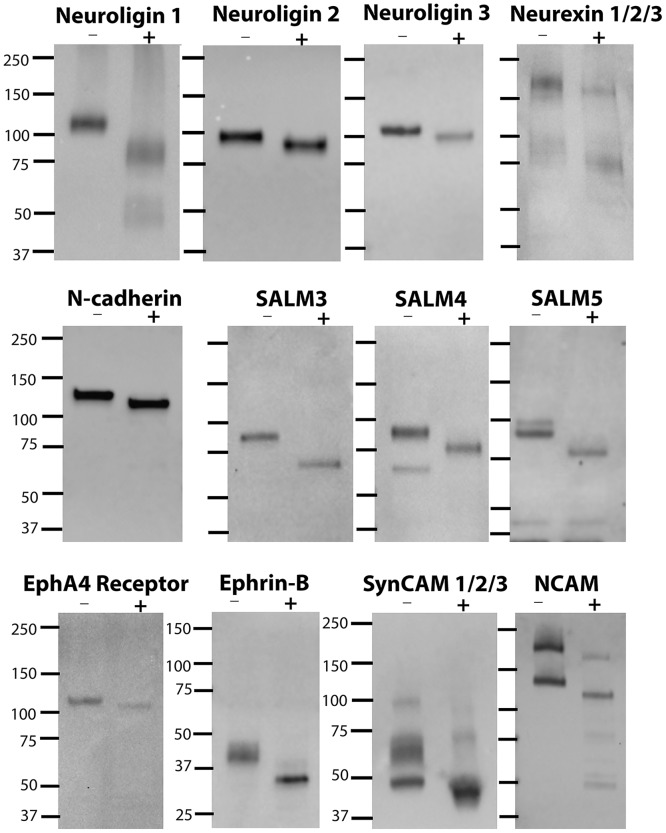
All tested cell adhesion molecules are glycosylated in the SJ fraction. Synaptic junction fractions were incubated with (+) or without (-) a cocktail of glycosidases (Sigma). All proteins tested in the SJ fraction show a shift in mobility after treatment with glycosidases.

After verification of the SJ fraction by electron microscopy and biochemistry, presence of a number of known and presumptive synaptic cell adhesion molecules in the SJ fraction was tested by Western immunoblotting. While the antibodies labeled one or more electrophoretic bands as expected, in most cases the labeled bands exhibited apparent molecular weights higher than predicted based on the proteins’ amino acid compositions. Since molecular weight is an important criterion for the identification of electrophoretic bands, we tested whether a post-translational modification is responsible for the discrepancy. Indeed, when SJ fractions were treated with a cocktail of glycosidases (Sigma, Enzymatic Deglycosylation Kit containing: PNGase F, Endo-O-Glycosidase, α-2(3,6,8,9)-Neuraminidase, β -(1,4)-Galactosidase, β-N-Acetylglycosaminidase) the apparent molecular weights of all labelled bands were reduced and corresponded more closely to the predicted molecular weights (Fig 4).

**Fig 5:**
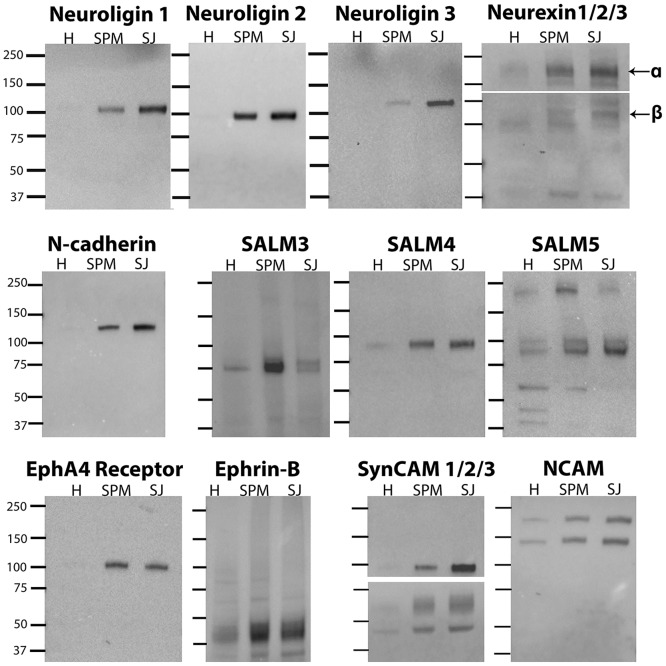
Some cell adhesion molecules are enriched in the SJ fraction, suggesting selective localization at the synaptic cleft. Western immunoblots comparing synaptic junction (SJ) fractions and parent homogenate (H) and synaptic plasma membrane (SPM) fractions. The lower portion of neurexin immunoblot and the upper portion of SynCAM 1/2/3 immunoblot correspond to higher exposure times to allow optimal visualization of all isoforms. Certain proteins including neuroligins, neurexins, N-cadherin, SynCAM 1 and SALM5 show distinctive enrichment. Equal amounts of protein were loaded into each lane. Experiments were repeated at least twice using different SJ fractions, with similar results (S2 Fig).

Fig 5 depicts Western immunoblots comparing levels of adhesion molecules in homogenate (H), SPM, and SJ fractions, using antibodies specific for the indicated proteins. All presumptive cell adhesion molecules tested exhibited enrichment in the SPM fraction compared to homogenate. However, only some of these proteins were also enriched in the SJ fraction compared to the parent SPM. Among those cell adhesion molecules enriched in the SJ fraction were all three isoforms (1, 2, 3) of neuroligins, and their binding partners neurexins (alpha- and beta-isoforms), as well as the homophilic cell adhesion molecule N-cadherin. On the other hand, fractionation of SALM family of proteins to the SJ was isoform-dependent. Levels of SALM3 decreased in the SJ fraction compared to those in the SPM fraction, while SALM5, and to a lesser degree SALM4, showed enrichment. Trans-synaptic binding partners, EphA4 receptor and ephrin-Bs exhibited decreased levels in the SJ fraction. One isoform of SynCAM with an apparent molecular weight of ~100kDa, which most likely corresponds to SynCAM 1 [16], showed marked enrichment in the SJ fraction compared to the SPM fraction. While the SynCAM isoforms with lower apparent molecular weights showed modest enrichment in the SJ fraction. Two main isoforms of NCAM also appeared to have a slight enrichment.

**Fig 6:**
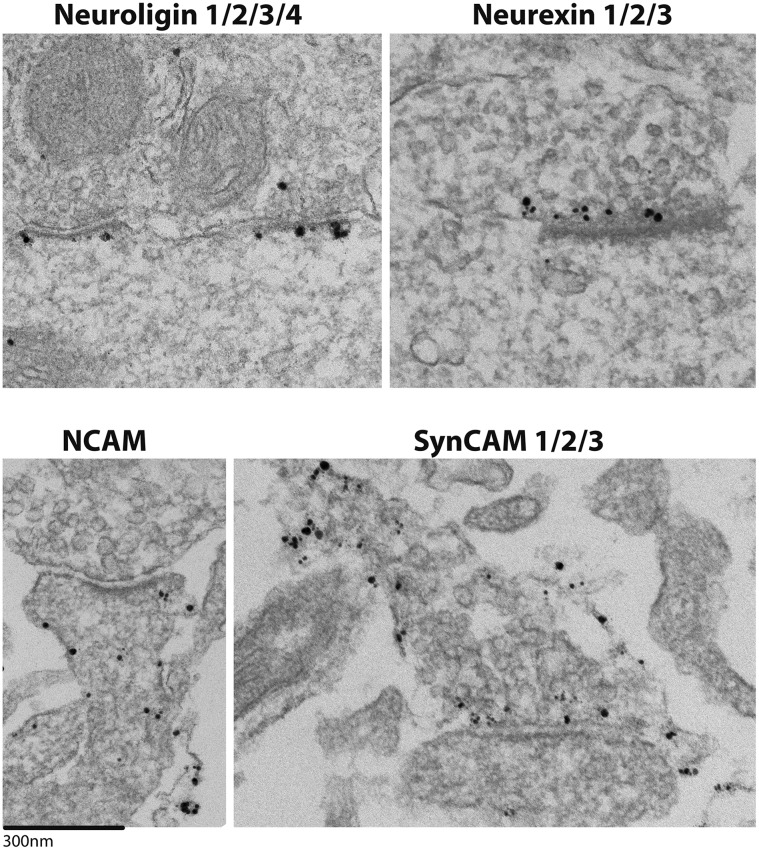
Localization of cell adhesion molecules by immuno-electron microscopy. Electron micrographs show synaptic regions from perfused mouse brain (top left image only) and cultured rat hippocampal neurons. Neuroligin and neurexin labeling is exclusive to the synaptic region, showing postsynaptic and presynaptic preferences respectively. Whereas, NCAM and SynCAM show a broader distribution within the neuron, with preferential labeling at dendritic and axonal plasma membranes, respectively.

Enrichment of a cell adhesion molecule in the SJ fraction compared to the parent SPM fraction is taken as a prediction of preferential localization at the synaptic cleft relative to other synaptic compartments. To verify findings from the Western immunoblotting experiments, pre-embedding immuno-electron microscopy was employed. Label for Neuroligin 1/2/3/4 (pan antibody) is localized selectively to the synaptic junctional area, specifically the postsynaptic membrane (Fig 6 top left). An antibody for neurexin 1/2/3 (pan), the trans-synaptic binding partner of neuroligin, also yields selective labeling at the synaptic junctional area, and, as expected, the label is at the presynaptic membrane (Fig 6 top right). These results are consistent with the enrichment of neuroligins and neurexins in the SJ fraction by Western immunoblotting. By contrast, the NCAM and SynCAM 1/2/3 labels show a broader distribution within the neuron. The label for NCAM is preferential to dendritic membranes (Fig 6 bottom left) with lesser labeling of axonal membranes (not shown). Whereas, the SynCAM 1/2/3 label is throughout the axolemma, including the synaptic junctional area (Fig 6 bottom right).

## Discussion

The present study describes a novel, detergent-free method for the preparation of a synaptic junction fraction. Ultrastructural observations indicate that treatment of conventional SPM fractions with phospholipase A_2_ to remove peripheral membranes yields relatively intact synaptic junctions. This method compares favorably to previously proposed strategies based on the use of low concentrations of mild detergents [10], [11], [12]. Selective digestion of peripheral membranes as compared to junctional membranes by phospholipase A_2_ is most likely due to differences in enzyme penetration. Indeed, junctional membranes lining the synaptic cleft are likely to be occluded by cleft material as well as by protein complexes lining the intracellular faces of membranes at the pre- and post-synaptic compartments. However, it should be noted that the synaptic cleft membranes are only less prone, but not immune, to phospholipase digestion. Thus, optimization of the reaction conditions, especially phospholipase concentration, through monitoring by electron microscopy is critical for the success of the preparation.

Removal of peripheral membranes upon digestion with phospholipase and centrifugation through a sucrose cushion should result in enrichment of proteins selectively localized at the synaptic junction. Comparison of the SJ fraction with parent H and SPM fractions shows marked enrichment in PSD-95, a protein localized almost exclusively at the postsynaptic density (Fig 2B top) [17], [18]. Additionally, mGluR5, a receptor shown by immuno-EM to preferentially localize to peripheral membranes [7], [8], [9], shows a significant decrease in the SJ fraction compared to the parent SPM fraction in agreement with the EM data. Thus, biochemical analysis of the SJ fraction complements observations by electron microscopy, indicating selective removal of peripheral membranes.

Further verification of the SJ fraction is provided by the enrichment of a group of well-studied synaptic cell adhesion molecules. Presynaptic neurexins are known to bind to postsynaptic neuroligins to form trans-synaptic complexes [19], [20]. Immuno-electron microscopy in intact neurons shows that label for neurexins and neuroligins localizes selectively to the synaptic junction to pre- and post-synaptic sides respectively of the synaptic cleft (Fig 6), [21], [22], [23], thus, in agreement with the results of biochemical analyses. Similarly, SJ fractions were enriched in N-cadherin, a protein that forms homophilic trans-synaptic bridges, in agreement with immuno-EM studies showing its selective localization at the synaptic junctional area [24], [25].

Among the family of synaptic adhesion-like molecules (SALMs), also known as Lrfns, only SALM5 exhibits a clear enrichment in the SJ preparation, while SALM3 levels decrease in the SJ preparation compared to the parent SPM fraction. Present results suggesting predominant SALM5 localization at the synaptic cleft are in agreement with the proposed function of this protein in regulating synapse development and AMPA-receptor mediated synaptic transmission [26]. Unfortunately, immuno-electron microscopy data for SALM5 is lacking due to the absence of a suitable antibody. Published immunogold labeling for SALM4 shows localizing at both the cleft and axonal membrane [27], consistent with the Western immunoblotting data showing only slight SALM4 enrichment in the synaptic junction fraction. The same study also mentions unpublished data indicating widespread distributions of SALM3 in the neuron [27], consistent with the decreased levels of these proteins in the synaptic junction fraction.

Western immunoblotting data finds decreased EphA4 receptor levels in the SJ fraction compared to the parent SPM fraction, suggesting this protein has a broader distribution, not limited to the synaptic cleft. These results are in agreement with previous immuno-EM and biochemical studies, showing that EphA4 is expressed in many neuronal compartments [28],[29]. Previous biochemical and immuno-EM studies of ephrin-Bs, however, reported localization at the PSD, [30], yet the data here shows a slight decrease in levels of ephrin-B (pan antibody) in the SJ fraction compared to parent SPM fraction. The differences may be due to different isoform specificities of antibodies used.

Western immunoblotting data shows a slight enrichment in two NCAM isoforms in the synaptic junction fraction compared to the parent SPM fraction. In contrast, our immuno-EM data shows that the label for NCAM avoids the synaptic junctional area and is distributed preferentially along dendritic membranes with some axolemmal distribution. The apparent discrepancy may be due to differences in antibody penetration, an issue that may be encountered in pre-embedding based techniques. Indeed, previous post-embedding immuno-EM studies show NCAM180 label selectively localized to postsynaptic densities [31], [32].

Results of biochemistry and immuno-electron microscopy, using the same (pan) antibody for SynCAM, are in agreement. Prominent enrichment in the SPM fraction compared to homogenate and slight enrichment in SJ fraction predict localization at synaptic junction as well as peripheral membranes. By EM, labeling is observed throughout the axolemma, including the junctional region. These results are in line with the proposal that SynCAMs are axon guidance molecules [33]. A higher molecular weight isoform of SynCAM, with a mobility ~ 100kDa shows more prominent enrichment in the SJ fraction. It is likely that this electrophoretic band corresponds to SynCAM 1 [16]. Interestingly, a recent article by Perez de Arce *et al* reported localization of SynCAM 1 at the postsynaptic edge [34].

Enrichment in the SJ fraction appears to be a good criterion for predicting selective localization of proteins at the synaptic cleft. Indeed, as discussed above, available immuno-electron microscopy data mostly verify predictions based on the biochemical analysis of the SJ fraction. In the present study, the new phospholipase A_2_-derived, SJ fraction was utilized for the identification of those cell adhesion molecules selectively located at the synaptic cleft. Similar experiments can be conducted to explore the distributions of neurotransmitter receptors, channels and other presumptive components of the synaptic cleft. Moreover, as illustrated by the deglycosylation experiments (Fig 4), the SJ preparation provides an in vitro experimental system for the assessment of post-translational modifications of proteins at the synaptic junction. The method developed here using tissue from cerebral cortex can also be applied for the preparation of SJ fractions from other regions of the mammalian brain, and maybe from other tissues as well, depending on the availability of a technique for the preparation of fractions enriched in synaptic structures, such as synaptosomes, and on a sufficiently occluded cleft structure that would limit penetration of phospholipase A_2_.

Availability of techniques for the isolation or enrichment of specific subcellular compartments are valuable tools for the identification and characterization of constituent proteins. Such preparations proved to be crucial in deciphering the molecular organization of postsynaptic densities (review: [35]) and synaptic vesicles [36]. We hope that the SJ preparation described here can fulfill a similar role for investigations on the molecular organization at the synaptic cleft.

## Supporting information

S1 Fig Assessments of four optimized synaptic junction preparations by electron microscopy. Every recognizable synaptic structure with a postsynaptic density (PSD) was counted and classified into one of the four categories (see Fig 2 for details). Individual data from four experiments are shown as blue, red, green and purple bars depicting percentages of each type of structure.
(TIF)

S2 Fig Additional experiments comparing levels of selected cell adhesion molecules in SJ and parent fractions. Western immunoblots comparing levels of proteins in H, SPM, and SJ fractions, using different SJ preparations from those used in Fig 5. The lower portion of the neurexin immunoblot and the upper portion of the SynCAM 1/2/3 immunoblot correspond to higher exposure times. Equal amounts of protein were loaded into each lane.
(TIF)
