# Supplementary figures and images for: A novel synaptic junction preparation for the identification and characterization of cleft proteins
Source: PLoS One. 2017 Mar 31;12(3):e0174895. doi: 10.1371/journal.pone.0174895 (PMC5376301; doi:10.1371/journal.pone.0174895)

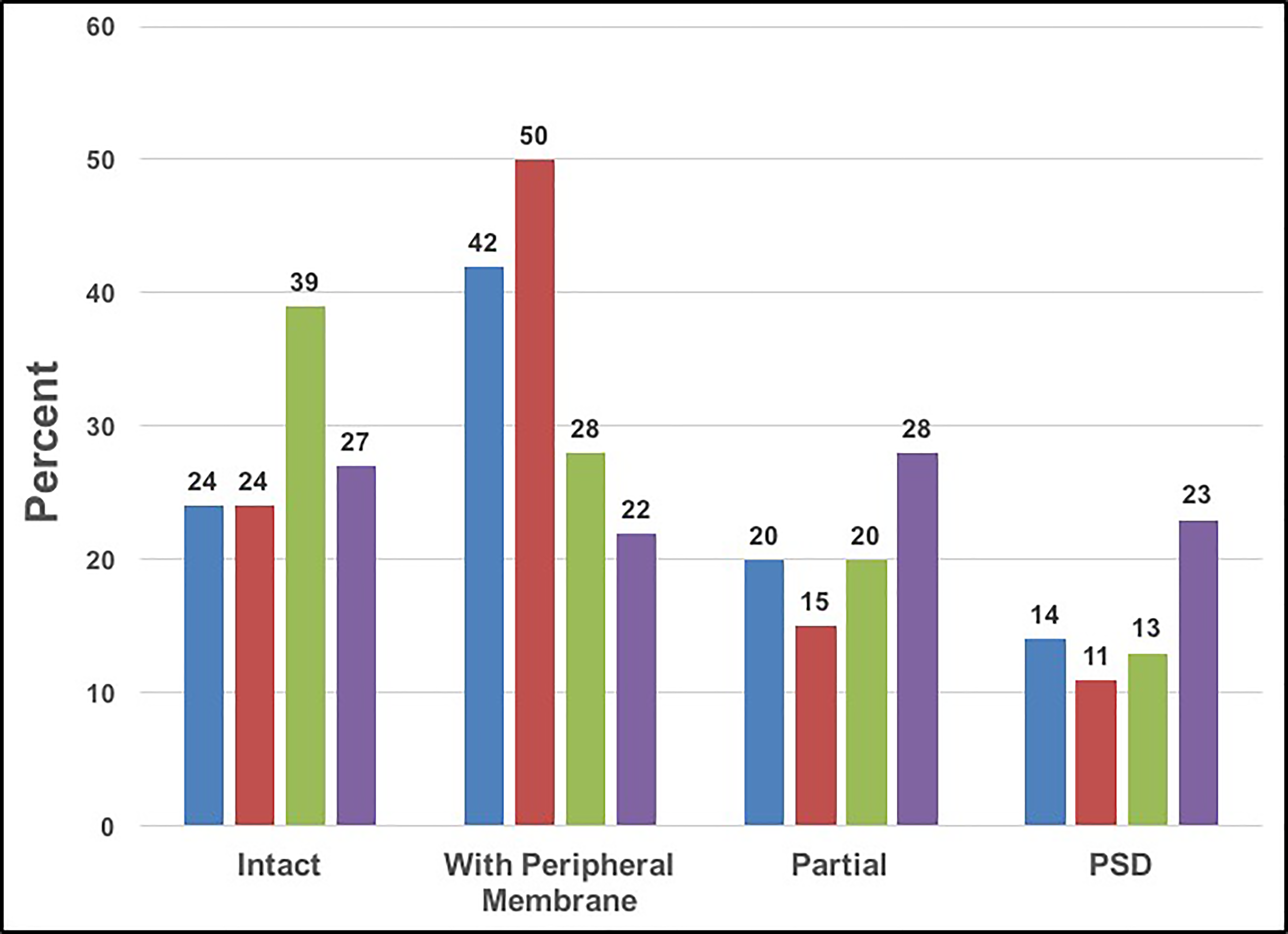

Supplement: S1 Fig — Every recognizable synaptic structure with a postsynaptic density (PSD) was counted and classified into one of the four categories (see Fig 2 for details). Individual data from four experiments are shown as blue, red, green and purple bars depicting percentages of each type of structure. (TIF) [file pone.0174895.s001.tif]

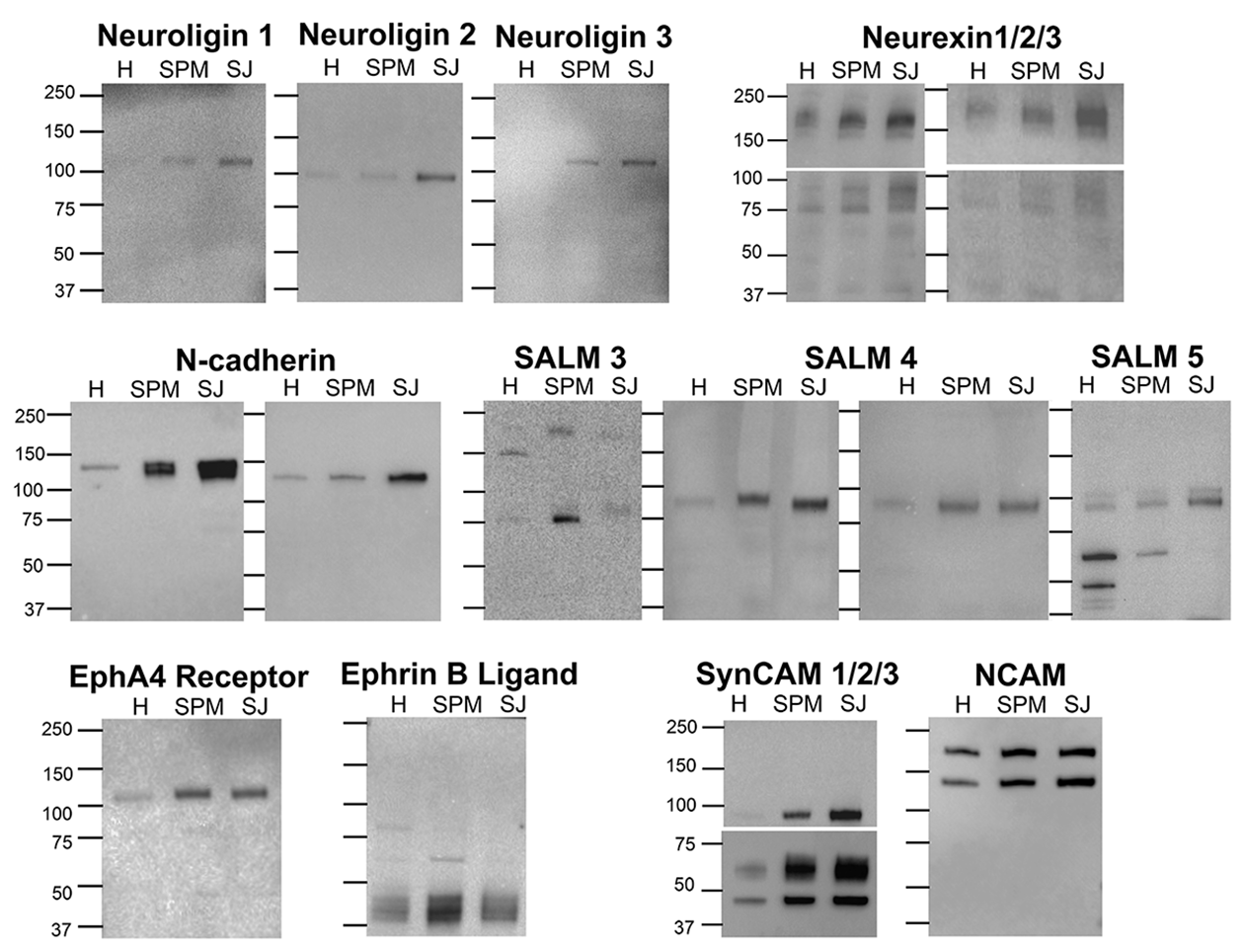

Supplement: S2 Fig — Western immunoblots comparing levels of proteins in H, SPM, and SJ fractions, using different SJ preparations from those used in Fig 5. The lower portion of the neurexin immunoblot and the upper portion of the SynCAM 1/2/3 immunoblot correspond to higher exposure times. Equal amounts of protein were loaded into each lane. (TIF) [file pone.0174895.s002.tif]
